# Psychiatric nurses’ knowledge of palliative care: current status and influencing factors

**DOI:** 10.3389/fpsyg.2025.1575655

**Published:** 2025-04-23

**Authors:** Cuixia Liu, Xiulan Liu, Xiashu Yan, Xiaodong Chen, Xucong He, Jian Liu, Tianyu Zeng

**Affiliations:** ^1^Department of Chronic Psychiatry, The Affiliated Brain Hospital, Guangzhou Medical University, Guangzhou, China; ^2^Key Laboratory of Neurogenetics and Channelopathies of Guangdong Province and the Ministry of Education of China, Guangzhou Medical University, Guangzhou, China; ^3^Geriatric Neuroscience Center, The Affiliated Brain Hospital, Guangzhou Medical University, Guangzhou, China; ^4^Department of Anesthesiology and Surgery, The First Affiliated Hospital of Guangzhou Medical University, Guangzhou, China

**Keywords:** psychiatric nurses, knowledge, palliative care, current status, factor

## Abstract

**Objective:**

This study aims to assess the current status of palliative care knowledge and identify the factors influencing it among healthcare workers in mental health institutions. The goal is to establish a scientific foundation for advancing palliative care practices in mental health settings.

**Methods:**

From August to September 2024, a survey was conducted to assess psychiatric nurses’ knowledge of palliative care. The study involved nurses from 47 mental health organizations in Guangdong Province, using general information and The Palliative Care Quiz for Nursing. Influencing factors were analyzed using one-way analysis of variance and multiple regression analysis.

**Results:**

A total of 625 psychiatric nurses completed valid questionnaires. The average score was 10.307 ± 3.305. Only 15.65% of nurses had attended palliative care training, while 29.39% demonstrated knowledge. It was significantly correlated with professional title (*r* = 0.164), marital status (*r* = 0.087), witnessing the death of a terminally ill patient or relative (*r* = 0.117), prior study (*r* = 0.164), willingness to engage in (*r* = 0.152), having children (*r* = 0.100), and performing cadaveric care (*r* = 0.086) (all with *p* < 0.05). Professional title (*B* = 0.497), academic qualification (*B* = 0.708), and witnessing the death of a terminally ill patient or relative (*B* = 0.932) significantly influenced it (all *p* < 0.05).

**Conclusion:**

Psychiatric nurses demonstrate limited awareness of palliative care, with a low percentage having received relevant training. However, there are a large number of long-term hospitalized psychiatric patients, it is crucial to provide systematic and specialized palliative care training for psychiatric nurses.

## 1 Introduction

Palliative care is designed to offer comprehensive support for patients with end-stage diseases or those in the final stages of life. This includes managing pain and discomfort, as well as providing physical, psychological, and spiritual care. The goal is to enhance the quality of life, ensuring that patients can experience their final days in comfort, peace, and dignity (National Health Office Family Letter, 2017). As China’s population ages, particularly during the 14th Five-Year Plan period, projections indicate that individuals aged ≥60 years will account for more than 20% of the total population, while the incidence of chronic diseases will continue to rise. To address this challenge, China’s “14th Five-Year Plan for Healthy Aging” explicitly outlines the need to advance the high-quality development of long-term care and palliative care services (National Health Aging Development, 2022).

The World Health Organization (WHO) reports that only 10% of the global population currently has access to palliative care. Given the aging population and the increasing prevalence of non-communicable diseases, the demand for palliative care is projected to double by 2060 (World Health Organization, 2021). This underscores the growing importance of palliative care in the global healthcare landscape.

However, palliative care in China is still in its early stages. Insufficient education and training remain the primary obstacles to its widespread implementation. Research indicates that much of the public’s understanding of palliative care comes from non-healthcare sources. Enhancing the education of healthcare professionals and leveraging their role in patient social networks could significantly boost public awareness and utilization of these services (Hicks-Courant et al., 2021). International best practices emphasize communication skills as a core component of palliative care education. Unfortunately, there is a lack of systematic research on palliative care education in China (Willemsen et al., 2021).

Furthermore, studies indicate that healthcare professionals urgently need training in communication and cultural sensitivity (Tay et al., 2021). Many patients with advanced cancer experience depression and anxiety, making the integration of psychosocial support into early palliative care essential for improving their mental health (Biswas et al., 2024). This further emphasizes the unique role psychiatric nurses play in palliative care.

Globally, approximately 1 billion people suffer from mental illnesses, many of whom also experience comorbid chronic conditions and face complex psychological and social challenges, making their health management more difficult. Patients with severe persistent mental illness (SPMI) bear a higher burden of chronic illness and are at greater risk of premature death. They also have limited access to palliative care due to delayed diagnoses and insufficient treatment options (Donald and Stajduhar, 2019). People diagnosed with a mental disorder had a higher mortality rate than the general population.All types of mental disorders are associated with shorter life expectancy (Plana-Ripoll et al., 2019). As these patients reach the end stages of their illness, their need for care intensifies, but healthcare system often struggles to meet these needs due to inequities in service delivery (Tinkler et al., 2021).

Psychiatry plays a crucial role in the healthcare system, with palliative care being a vital component of mental health services. As the primary providers of psychological support, psychiatric nurses significantly impact the quality of palliative care. Studies have shown that the current palliative care model often fails to meet the unique needs of patients with mental illnesses. This highlights the need for greater integration of psychiatry and palliative care services, along with improved professional training and interdisciplinary collaboration among healthcare providers (Shalev et al., 2024).

Exploring the current state of psychiatric nurses’ knowledge of palliative care, as well as the factors influencing it, is crucial not only for improving the palliative care service system but also for providing a practical foundation for future nursing training and the planning of psychiatric palliative care units.

## 2 Materials and methods

### 2.1 Subjects

A questionnaire survey was conducted between August and September 2024, targeting nurses in 47 mental health organizations across Guangdong Province. The inclusion criteria were as follows: (1) registered nurses in Guangdong Province; (2) at least one year of experience working in psychiatric wards; (3) voluntary participation. Exclusion criteria included: (1) serious physical illness, psychological conditions, or cognitive disorders; (2) participants currently separated from the workplace. This study obtained ethical approval from the IRB of the Affiliated Brain Hospital, Guangzhou Medical University (approval number: 2024076), and written informed consent was obtained from all participants.

### 2.2 Questionnaire

#### 2.2.1 General information

The survey gathered general demographic information, including gender, age, religious beliefs, academic qualification, professional title, years of experience, marital status, whether the participant had children, witnessed the death of a terminally ill patient or relative, experienced in caring for terminally ill patients in the past year, involvement in cadaveric care during the last year, studied about palliative care, and whether the participant currently works in a palliative care department or role. Additionally, it asked whether the participant was willing to engage in palliative care services.

#### 2.2.2 The Palliative Care Quiz for Nursing (PCQN)

The Palliative Care Quiz for Nursing is a tool to measure the palliative care knowledge of nurses. The questionnaire was developed by Ross et al. (1996). In this study, the Chinese version of the Palliative Care Quiz for Nursing, translated by Zou (2007), was employed. This adaptation underwent meticulous cultural refinement and rigorous validation processes to ensure its relevance and appropriateness for Chinese nurses.

It comprised 20 questions across three dimensions. The questions were of a judgmental nature and were categorized as “correct,” “incorrect,” or “do not know.” Correct answers were scored 1 point, while incorrect answers and “do not know” responses were scored 0 points. The maximum possible score was 20, with higher scores indicating greater knowledge. The Cronbach’s alpha coefficient for the scale was 0.765, demonstrating good internal consistency and reliability.

### 2.3 Data collection

The Palliative Care Quiz for Nursing was uploaded to Questionnaire Star, with detailed instructions provided on the landing page. These instructions emphasized that participation was voluntary and that all responses would remain anonymous and confidential. Additionally, the study’s purpose, significance, and guidelines for completing the questionnaire were explained to ensure participants could respond thoughtfully. Participants were also informed that they could withdraw from the survey at any time.

To prevent duplicate submissions, each device was restricted to a single login. To ensure completeness, the survey included a breakpoint resume function, allowing participants to complete all questions before submitting their responses. The researcher carefully reviewed each returned questionnaire, identifying and eliminating those with abnormal completion times or responses showing clear patterns. A total of 635 questionnaires were collected, of which 625 were valid, resulting in an effective response rate of 98.4%.

### 2.4 Sample size

Using the formula, sample size = [max (variable) × (5–10)] × [1 + (15–20%)], with 17 variables included in this study and accounting for a 15% lapse, the sample size was estimated to be between 98 and 204. Given that this was a multicenter study with a broad sample range, the final sample size reached 635. All participants met the inclusion and exclusion criteria.

### 2.5 Statistical analysis

Data analysis was conducted using IBM SPSS 21.0. Categorical data were summarized using frequency and percentage distributions, while continuous data were expressed as means ± standard deviations. Group differences were assessed using independent samples t-tests and one-way analysis of variance (ANOVA). Correlations were examined via Pearson correlation analysis, and influencing factors were identified through multiple regression analysis. Statistical significance was set at *p* < 0.05.

## 3 Results

### 3.1 Level of palliative care knowledge

The mean score for palliative care knowledge was 10.307 ± 3.305. The scores for each item are shown in Table 1.

**Table 1:** Scores for each item in the Palliative Care Quiz for Nursing (Total items = 20).

	Item	Correct	Incorrect
1	Palliative care is only suitable for patients whose condition is deteriorating or worsening.	286 (45.8%)	339 (54.2%)
2	Morphine is the reference standard for the pain-relieving effects of other opioid drugs.	548 (87.7%)	77 (12.3%)
3	The course of the disease determines the method of pain treatment.	90 (14.4%)	535 (85.6%)
4	Complementary therapies are important for pain control.	526 (84.2%)	99 (15.8%)
5	It is crucial for family members to be at the patient’s bedside until death.	32 (5.1%)	593 (94.9%)
6	In the final stages of a patient’s life, drowsiness associated with electrolyte imbalances reduces the need for sedation.	517 (82.7%)	108 (17.3%)
7	The main problem faced with long-term use of morphine for pain relief is drug addiction.	70 (11.2%)	555 (88.8%)
8	Patients taking opioids should also be given bowel treatment. (That is, preventive measures and treatment for gastrointestinal symptoms.)	217 (34.7%)	408 (65.3%)
9	Providing palliative care requires emotional detachment.	50 (8.0%)	575 (92.0%)
10	Medications that cause respiratory depression are appropriate for the treatment of severe respiratory distress in the terminal stage of a disease.	397 (63.5%)	228 (36.5%)
11	Men generally can alleviate their sadness faster than women.	482 (77.1%)	143 (22.9%)
12	The concept of palliative care is consistent with the concept of active treatment.	477 (76.3%)	148 (23.7%)
13	The use of placebos is appropriate in treating certain types of pain.	89 (14.2%)	536 (85.8%)
14	High doses of codeine are more likely to cause nausea and vomiting than morphine.	401 (64.2%)	224 (35.8%)
15	Pain and physical pain are synonyms.	524 (83.8%)	101 (16.2%)
16	Dilaudid is not an effective pain reliever for chronic pain.	288 (46.1%)	337 (53.9%)
17	The cumulative feeling of loss from caring for dying patients inevitably leads to physical and emotional exhaustion in palliative care staff.	172 (27.5%)	453 (72.5%)
18	The clinical manifestations of chronic pain are different from those of acute pain.	519 (83.0%)	106 (17.0%)
19	Losing distant relatives or those with whom one has a poor relationship is easier to cope with than losing close relatives or those with whom one has a close bond.	536 (85.8%)	89 (14.2%)
20	Fatigue or anxiety can cause a decrease in pain threshold.	221 (35.4%)	404 (64.6%)

### 3.2 Factors influencing the level of palliative care knowledge

#### 3.2.1 Demographic characteristics and current perceptions of palliative care

Among the 625 psychiatric nursing staff, 153 (24.5%) were male, and 472 (75.5%) were female, with a mean age of 34 ± 8 years. 439 (70.2%) were married, 583 (93.3%) reported no religious beliefs, 397 (63.5%) held an undergraduate degree or higher, and 282 (45.1%) held a professional title of intermediate level or above. Only 15.65% of nurses had attended palliative care training, while 29.39% demonstrated knowledge and 26.84% expressed willingness to work in palliative care.

#### 3.2.2 One-way ANOVA of psychiatric nurses’ knowledge of palliative care

One-way ANOVA revealed significant differences in palliative care knowledge based on gender, age, years of experience, academic qualification, professional title, parental status, witnessing the death of a terminally ill patient or relative, recent cadaveric care involvement, prior palliative care education, and willingness to engage in palliative care services (*p* < 0.05), as shown in Table 2.

**Table 2:** One-way ANOVA of psychiatric nurses’ knowledge of palliative care (*n* = 625).

Item	Frequency	Mean ± standard deviation	*F/t*	*P*
Sex			−2.259	0.024
male	153	9.78 ± 4.12		
female	472	10.48 ± 2.98		
Age			2.656	0.048
≤30 years old	243	9.81 ± 3.72		
31–40 years old	245	10.62 ± 3.15		
41–50 years old	111	10.53 ± 2.77		
≥51 years old	26	10.33 ± 1.53		
Working experience			4.023	0.018
1–5 years	147	9.97 ± 3.28		
6–10 years	183	9.94 ± 3.83		
11 years	295	10.70 ± 2.91		
Academic qualification			8.750	<0.000
Secondary and below	39	8.95 ± 4.02		
Junior college	189	9.56 ± 3.55		
Undergraduate	390	10.78 ± 3.01		
Postgraduates	7	11.57 ± 1.81		
Professional title			6.940	<0.000
Staff nurse	129	9.24 ± 3.84		
Nurse practitioner	214	10.08 ± 3.76		
Charge nurse	254	10.91 ± 2.50		
Associate chief nurse	26	11.42 ± 1.27		
Chief nurse	2	12.50 ± 0.71		
Marital status			2.099	0.099
Single	168	9.76 ± 3.63		
Married	439	10.51 ± 3.14		
Divorced	16	10.44 ± 3.78		
Widowed	2	10.50 ± 3.54		
Children			−2.664	0.008
No	225	9.84 ± 3.54		
Yes	400	10.57 ± 3.14		
Religious belief			0.817	0.414
No	583	10.34 ± 3.31		
Yes	42	9.90 ± 3.24		
Witnessed the death of a terminally ill patient or relative			3.783	<0.000
No	148	9.42 ± 3.98		
Yes	477	10.58 ± 3.02		
Carried out care for terminally ill patients in the last year			−1.273	0.203
No	410	10.19 ± 3.36		
Yes	215	10.54 ± 3.19		
Performed cadaveric care in the past year			−2.770	0.006
No	483	10.11 ± 3.49		
Yes	142	10.98 ± 2.47		
Studied about palliative care			4.896	<0.000
No	183	11.2951 ± 2.03		
Yes	442	9.8982 ± 3.63		
Working in a palliative care unit			1.426	0.154
No	582	10.26 ± 3.36		
Yes	43	11.00 ± 2.39		
Willing to engage in palliative care services			3.678	<0.000
No	457	10.02 ± 3.48		
Yes	168	11.10 ± 2.62		

Specifically, females demonstrated higher palliative care knowledge than males. Nurses aged 31–40 years, with more than 11 years of experience, holding an undergraduate degree or higher, and an intermediate or higher professional title, scored significantly higher. Additionally, nurses with children also exhibited higher levels of palliative care knowledge. Furthermore, those who had witnessed the death of a terminally ill patient or relative, had performed cadaveric care in the past year, had studied palliative care-related knowledge, and were willing to engage in palliative care services showed higher scores in palliative care knowledge, as shown in Table 2.

#### 3.2.3 Correlation analysis of palliative care knowledge among psychiatric nurses

Correlation analysis showed that palliative care knowledge was positively correlated with professional title, marital status, cadaveric care experience, witnessed the death of a terminally ill patient or relative, prior study of palliative care, willingness to engage in palliative care services, and openness to providing contact information for follow-up interviews. Psychiatric nurses with relevant experience and willingness to participate in palliative care had significantly higher palliative care knowledge, as shown in Tables 3, 4.

**Table 3:** Variable assignment for the correlation analysis of palliative care knowledge among psychiatric nurses.

Variables	Assignment description
Independent variable	
Professional title	1 = Staff nurse, 2 = Nurse practitioner, 3 = Charge nurse, 4 = Associate chief nurse, 5 = Chief nurse
Marital status	1 = single, 2 = married, 3 = divorcee, 4 = widowed
Witnessed the death of a terminally ill patient or relative	0 = no, 1 = yes
Studied about palliative care	0 = no, 1 = yes
Willingness to engage in palliative care services	0 = no, 1 = yes
Leave your contact information for a follow-up interview	0 = no, 1 = yes
Have children	0 = no, 1 = yes
Performed cadaveric care in the past year	0 = no, 1 = yes

**Table 4:** Correlation analysis of palliative care knowledge among psychiatric nurses (r).

Variables	Total palliative knowledge score	Professional title	Marital status	Witnessed a terminally ill patient or relative	Studied about palliative care	Willingness to engage in palliative services	Leave your contact information for a follow-up interview	Children	Performed cadaveric care in the past year
Total palliative knowledge score									
Professional title	0.164**								
Marital status	0.087*	0.533**							
Witnessed a terminally ill patient or relative	0.117**	0.162**	0.121**						
Studied about palliative care	0.164**	0.082*	0.045	−0.044					
Willingness to engage in palliative services	0.152**	−0.016	0.025	−0.100*	−0.316**				
Leave your contact information for a follow-up interview	0.125**	0.009	0.051	−0.044	−0.06	−0.342**			
Children	0.100*	0.543**	0.772**	0.100*	−0.008	−0.034	0.058		
Performed cadaveric care in the past year	0.086*	0.099*	0.073	0.185**	0.096*	0.162**	0.085*	0.033	

#### 3.2.4 Multifactorial logistic regression of psychiatric nurses’ knowledge of palliative care

Y (Total score for palliative care knowledge) = 8.492 + 0.497 × (professional title) + 0.708 × (academic qualification) + 0.932 × (witnessed a terminally ill patient or relative), as shown in Tables 5, 6.

**Table 5:** Multifactorial logistic regression variables assigned to psychiatric nurses’ knowledge of palliative care.

Variables	Assignment description
Independent variable	
Professional title	1 = Staff nurse, 2 = Nurse practitioner, 3 = Charge nurse, 4 = Associate chief nurse, 5 = Chief nurse
Academic qualification	1 = Secondary and below, 2 = junior, 3 = undergraduate, 4 = postgraduate
Witnessed a terminally ill patient or relative	0 = no, 1 = yes

**Table 6:** Multifactorial logistic regression of psychiatric nurses’ knowledge of palliative care.

Variables	*B*	SE	β	t	*P*
(constant)	8.492	0.705	-	12.04	0
Professional title	0.497	0.169	0.128	2.939	0.003
Academic qualification	0.708	0.228	0.134	3.107	0.002
Witnessed a terminally Ill patient or relative	0.932	0.304	0.12	3.063	0.002

*R = 0.266; R2 = 0.071; adapt R2 = 0.066; F = 15.808983; p < 0.01.*

* Significant correlation at the 0.05 level (two-tailed). ** Significant at the 0.01 level (two-tailed).

## 4 Discussion

### 4.1 Low overall level of palliative care knowledge among psychiatric nurses

Psychiatric nurses’ overall palliative care knowledge was low (mean score: 10.307 ± 3.305), consistent with Zou Min’s findings (Zou, 2015). This reflects a significant gap in psychiatric nurses’ understanding of palliative care.

In 2017, China introduced the “Palliative care Practice Guideline (for Trial Implementation)” (Medical Affairs Bureau, 2017), which established standards for assessment, operational procedures, and precautions. This marked an important step in recognizing the role of palliative care in China. Similarly, the “Clinical Practice Guidelines for Palliative Care in the 12 Months Before Dying,” published by the Registered Nurses’ Society of Ontario, Canada, in 2020 (Wang et al., 2022), provides critical guidance for enhancing palliative care knowledge.

Elderly psychiatric patients often suffer from long-term chronic illnesses and comorbidities, increasing their need for palliative care. However, psychiatric nurses prioritize symptom management and psychological support over palliative care concepts. Currently, palliative care is predominantly utilized in oncology, intensive care units (ICUs), and other specialty departments, where nurses have a high demand for palliative care knowledge (Luo et al., 2021). In contrast, psychiatric nurses have fewer opportunities to engage with terminally ill patients, leading to a neglect of their need for palliative care knowledge.

A survey by Xu et al. (2023) involving 1,819 nursing staff across 45 hospitals and nursing homes in Hainan Province revealed that nursing staff held a moderately approving attitude toward palliative care and demonstrated a strong desire for further training. These findings highlight the need for administrators to foster nurses’ intrinsic motivation and reform the palliative care training system (Ma et al., 2021), strengthening psychiatric nurses’ knowledge and training in palliative care. Additionally, an increased focus on life and death education (Teng et al., 2021) is essential to enhancing nurses’ competence in palliative care.

### 4.2 Factors influencing psychiatric nurses’ perspectives

Several factors influenced palliative care knowledge, including gender, age, parental status, academic qualification, professional title, years of experience, witnessing the death of a terminally ill patient or relative, performing cadaveric care in the last year, prior study of palliative care, and willingness to engage in palliative care services.

First, gender, age, childbearing, among personal characteristics had a significant effect on palliative care. As psychiatric nurses age, they encounter a broader range of patients, particularly elderly and chronically ill individuals. This clinical experience contributes to the gradual acquisition of core palliative care knowledge as nurses progress in their practice.

Furthermore, older nurses are more likely to participate in professional training organized by their institutions. They tend to be more emotionally mature and stable, which allows them to better understand and respond to the emotional fluctuations and behavioral changes of both patients and their families. Effective communication is a crucial component of this competency framework (White et al., 2021), as it plays a key role in alleviating patient and family suffering (Wang, 2017; Li et al., 2023) and is indispensable in the implementation of palliative care.

Childbearing was a significant factors influencing the level of palliative care knowledge, a result that differs from the findings of Tian et al. (2018). This discrepancy may be explained by the fact that nurses with children tend to have a more profound understanding of key palliative care concepts—such as the meaning of life, family support, and end-of-life care—due to their family roles. Particularly, parents often assume pivotal supportive and caregiving responsibilities within the family. As a result, they are more attuned to emotional dynamics, family relationships, and the value of life, which motivates them to actively seek out knowledge and excel in palliative care.

In addition to personal characteristics, professional experience is an important factor in the level of palliative care knowledge.

Psychiatric nurses with advanced degrees and professional title typically demonstrate superior palliative care knowledge, consistent with findings by Teng et al. (2021). These nurses combine strong theoretical foundations with extensive clinical experience, exhibiting both professional excellence and commitment to humanistic care (Hu et al., 2023). Their advanced education facilitates access to palliative care literature, while their clinical expertise enhances theoretical understanding. Furthermore, they prioritize continuous learning, regularly updating their knowledge to maintain clinical competence. These combined factors contribute to their advanced palliative care proficiency.

Notably, relevant clinical experiences significantly influenced palliative care knowledge levels. Exposure to terminal patient deaths, participation in cadaveric care, and palliative care training all substantially improved nurses’ understanding of palliative care. These hands-on experiences provided intuitive insights into palliative care’s importance while reinforcing conceptual knowledge and practical skills (Li et al., 2023; Tian et al., 2018).

Nurses involved in palliative care practice or training gain a deeper understanding of palliative care concepts and skills through exposure to diverse cases. Such experiences heighten their appreciation of palliative care’s significance and foster ongoing professional development. Shu et al. (2022) demonstrated that even short-term educational interventions could effectively enhance medical staff’s palliative care knowledge and skills. These findings underscore the value of implementing diverse educational strategies to enhance medical staff’s knowledge and skills, while promoting greater engagement in palliative care services.

Furthermore, psychiatric nurses with a passion for palliative care typically dedicate more time and effort to learning about it, resulting in higher levels of knowledge. The WHO reported in October 2021 that to achieve optimal palliative care at the national level, countries must establish supportive policy environments, empower communities, conduct palliative care research, build robust education and training systems for palliative care professionals, and prioritize the quality of palliative care services (World Health Organization, 2021). With this infrastructure, more nurses are likely to engage in palliative care.

### 4.3 Limitation

Although this study provides valuable insights into the level of palliative care knowledge among psychiatric nurses and the factors that influence it, limitations remain. The sample was derived from psychiatric nurses in one region only, which may be geographically and institution-specific, limiting the generalizability of the findings. Future studies could expand the sample to cover different regions and types of healthcare organizations to improve the representativeness of the results.

## 5 Summary

This study reveals that the overall level of palliative care knowledge among psychiatric nurses is relatively low, highlighting a critical gap in their professional competencies. Personal characteristics (e.g., gender and age), professional experiences (e.g., academic qualification and professional title), and relevant experiences (e.g., witnessing the deaths of terminally ill patients and performing cadaveric care significantly influenced palliative care knowledge). These results suggest the need for targeted interventions to enhance psychiatric nurses’ understanding and application of palliative care knowledge.

Studies have indicated significant growth in research on palliative care needs in China, with the field now entering an exploratory phase (Chu and Chen, 2024). As such, hospital management should prioritize strengthening palliative care knowledge training for psychiatric nurses, offering increased learning and practical opportunities. Special attention should be given to young nurses and those with no prior experience in palliative care to enhance their knowledge in this area. Additionally, fostering nurses’ interest in palliative care practice and providing career development support are crucial strategies for improving palliative care knowledge.

## Data Availability

The original contributions presented in the study are included in the article, further inquiries can be directed to the corresponding author.
